# Human rights violations among sexual and gender minorities in Kathmandu, Nepal: a qualitative investigation

**DOI:** 10.1186/1472-698X-12-7

**Published:** 2012-05-16

**Authors:** Sonal Singh, Sunil Babu Pant, Suben Dhakal, Subash Pokhrel, Luke C Mullany

**Affiliations:** 1 Center for Public Health and Human Rights, Johns Hopkins Bloomberg School of Public Health, Baltimore, USA; 2 Department of International Health, Johns Hopkins Bloomberg School of Public Health, Baltimore, USA; 3 Department of Medicine, Johns Hopkins University School of Medicine, Baltimore, USA; 4 Blue Diamond Society, Kathmandu, Nepal

## Abstract

**Background:**

Nepal has experienced sporadic reports of human rights violations among sexual and gender minorities. Our objective was to identify a range of human rights that are enshrined in international law and/or are commonly reported by sexual and gender minority participants in Kathmandu, to be nonprotected or violated.

**Methods:**

In September 2009 three focus group discussions were conducted by trained interviewers among a convenience sample of sexual and gender minority participants in Kathmandu Nepal. The modified Delphi technique was utilized to elicit and rank participant-generated definitions of human rights and their subsequent violations. Data was analyzed independently and cross checked by another investigator.

**Results:**

Participants (n = 29) reported experiencing a range of human rights violations at home, work, educational, health care settings and in public places. Lack of adequate legal protection, physical and mental abuse and torture were commonly reported. Access to adequate legal protection and improvements in the family and healthcare environment were ranked as the most important priority areas.

**Conclusions:**

Sexual and gender minorities in Nepal experienced a range of human rights violations. Future efforts should enroll a larger and more systematic sample of participants to determine frequency, timing, and/or intensity of exposure to rights violations, and estimate the population-based impact of these rights violations on specific health outcomes

## Background

With a population of more than 29 million people, Nepal is one of the poorest countries in South-Asia [1] A decade-long insurgency has weakened social and physical infrastructure in Nepal [2-4]. Sexual and gender minorities in Nepal, including men who have sex with men and third gender people, constitute a vulnerable population with limited legal protections in Nepal [5,6]. Nepal’s ratification of several human rights treaties, including the Convention against Torture and other Cruel, Inhuman or Degrading Treatment or Punishment, the International Covenant on Civil and Political Rights, and the International Covenant on Economic Social and Cultural Rights, has not translated into concrete human rights protection for sexual and gender minorities [4].

There have been anecdotal reports of human rights violations among sexual and gender minorities in Nepal [5-7]. Violence towards Metis (cross-dressing effeminate males) by law enforcement officers, including rape, has been reported, and metis may be potentially targeted because of their feminine gender presentation [5]. These reports, however, have usually been secondary accounts of sporadic incidents, and systematic assessments of the range of human rights violations faced by sexual and gender minorities are lacking.

Men who have sex with men in Nepal also have high rates of HIV [8,9]; increased risk among men who have sex with men and third genders in Nepal is driven by marginalization in a traditional society. Human rights violations have the potential to be linked to poor health outcomes, including mental and sexual health outcomes. Our objective was to identify a range of human rights that are enshrined in international law and/or are commonly reported by sexual and gender minority participants, especially men who have sex with men and third genders in Kathmandu, to be nonprotected or violated.

## Methods

Over 3 consecutive days in September 2009, our local community partners from the Blue Diamond Society, an organization for sexual and gender minorities in Nepal, telephonically invited a convenience sample of 10 men who have sex with men, third genders and transgender people from the tri-city metropolitan area of Kathmandu to participate in daily discussion group sessions (n = 30). Participants were chosen purposively to represent socio-economic and geographic areas of Kathmandu. The participants were informed that their discussion would inform the development of a health and human rights scale for men who have sex with men and third gender people. In order to maintain the confidentiality and anonymity of our participants we did not collect individual demographic information or data related to sexual behaviour or identity on the participants. These sessions were held at a safe and secure location that ensured anonymity and confidentiality of participants. A standardized written protocol adapted from the modified Delphi technique was followed for the conduct of this study [10,11].

After obtaining written informed consent from each of the participants, written summaries of international human rights documents including the international covenant on civil and political rights and the international covenant on social, economic and cultural rights were provided to the participants in Nepali. We also provided them a summary and tentative list of potential human rights violations developed by our community partners to initiate discussion. The participants were entrusted with generating and ranking a *participant-generated* list of *human rights violation*s relevant to sexual and gender minorities in Nepal based on their experiences. Two study investigators acted as facilitators for the discussion (SS and MB), while a third facilitator (SP) maintained a running list of specific rights violations generated in the discussion. As participants shared their experiences, the initial list was updated and refined. All participants were given the opportunity to contribute, with follow up comments and conversation encouraged until saturation of ideas and specific descriptions of violations had been achieved. After generating a complete list on a whiteboard, the participants reviewed the violations in a group and assigned categories such as those experienced at the household level, neighborhood level, schools, public places, health care settings and violations at the hands of authorities. Apart from these specific domains, the participants elaborated on additional domains and physical and mental health issues associated with rights violations or as a consequence of these violations.

Lastly, each participant independently and blinded to the choices of others in the group assigned numerical rankings to their top 3 choices of categories of human rights violations perceived to be of greatest importance to their daily lives. The individual rankings were tallied over all participants in the group to identify the top three categories i.e. those ranked by the highest number of participants, based on the preferred ranking by the most number of participants. In the event of a tie the choices selected in the next highest category were evaluated as a tie-breaker. The top three choices were reviewed by the group to ensure that the rankings reflected their priorities.

These discussions were conducted in morning sessions lasting approximately four hours. Identical procedures were followed on each of the three days. Each focus group discussion was followed by a debriefing session among the investigator team in the afternoon to ensure that those examples of rights violations that had not been discussed earlier were explored on the next day. Care was taken to avoid contamination of ideas from the previous discussion by ensuring that each participant had an opportunity to report experiences relevant to all the domains. The facilitators guided the discussed and clarified concepts as an iterative and interactive process. None of the participants received any remuneration. The participants were identified by numeric identifiers only and no identifiable information was recorded. All discussions were conducted in Nepali. The sessions were not audio recorded to protect the participants. Two additional experienced note takers (n = 6 note takers) made daily notes in Nepali and translated them into English. SS and MB used content analyses in examining the data [12]. Themes derived from the focus group discussions were grouped into categories: 1) a general category denoting broad description of participant generated listing of human rights violations; 2) location-specific categories including within-household, in educational settings, at the workplace, in public places representing the life-course of these participants from more private to more public settings and; 3) context specific categories including violations at the hands of the authorities and violations due to the lack of adequate legal protections.

### Human participant protection

Ethical approval for the study was provided by the Institutional Review Board of the Nepal Health Research Council, Kathmandu, Nepal and the Johns Hopkins Bloomberg School of Public Health, Baltimore, USA.

## Results

Among the 30 individuals invited to participate, one participant was unable to attend. The 29 included participants represented different ethnic groups, ranged in age from 18 to 55 years, and included bisexual, men who have sex with men, third genders, and *metis* (cross-dressing effeminate males). Some were married, and lived with family, while others were single living alone or with their partners. They represented different socioeconomic classes, occupations (sex workers, government employees, students), and residence status (local residents, migrants from India, some displaced as a result of the conflict). All participants reported at least one personal experience with human rights violations during the last few years. We present selected representative verbatim quotes from the participants and parenthetical explanation for unfamiliar words.

### What are human rights violations?

Participants’ responses to the initial query of broad conceptualization of human rights demonstrate identification with both physical and structural constructs of rights violations. One participant described human rights violations as:

"*“Violations of the rights I was born with.”*"

Another participant elaborated:

"“[Human Rights Violations are] *not only gross physical violation but also subtle violations about how we are treated and how we feel.”*"

They offered specific examples

"*“We [Men who have Sex with Men] can’t get jobs, our families throw us out. We are forced into sex work, but then the police detain us. How will we survive? “*"

Several participants stressed the importance of: “*gaas, bas ra kapas* [food, shelter and clothing]” as being fundamental human rights. According to another participant human rights are: *“the right not to be treated like animals* “

### Human rights violations in the household

Several participants reported experiencing rights violations in the household because of their sexual inclination or their preference to neither marry nor raise children. One participant described:

*“I married 15 yrs ago under marriage pressure. My family discriminated against me and denied me my rightful property rights. Subsequently, my wife left me. My daughters get teased at school because of my* [Men who have Sex with Men] *status. The Newari community has shunned me and don’t invite me to the Guthi* [a social organization or a patriarchial kinship used to maintain social order among the Newars]. *I have not been back home in 5 years.”*

Another participant echoed his exclusion from cultural ceremonies.

"*“I was not allowed to attend Bartaman* [the sacred thread ceremony which is a traditional rite of passage for Brahmin boys] *for my uncle’s son. “*One participant stated, “*Our families have disinherited us and would not come to bury us when we die.”*"

The participants reported experiencing human rights violations at the hands of parents, siblings, and children. Several participants chose not to disclose their status to their families in anticipation of the potential household and family consequences arising from knowledge of their sexual identity and inclination. A few participants who supported their family through their work reported experiencing no rights violations at home. It is possible that their employment status and role within the household related to earnings and support may influence this relationship.

### Human rights violations in school and workplace

Several participants reported being denied entrance to educational institutions because of the lack of specific laws. A participant stated,

"*“It is hard for us to get admitted to school because our names are like boys but our photographs are like girls. We get called names like ‘chakka’* [derogatory slang for a homosexual or gay]. *In the context of a class of gender when I questioned the teacher about third gender/intersex, the teacher was upset and removed me from class.”*"

Several participants reported being approached for sexual favors. *“One of the other sirs was drunk and asked me to come with him. He said ‘You make out with one sir, why didn’t you have sex with me?’ The teachers failed me in school because I did not have sex with them.”*

Some participants described being offered lower salaries, being denied vacation time or being teased by coworkers because of their gait or shrill voice. One participant described:

*“I was a dancer at a Casino. I was removed from work because I was a Hijra* [males who adopt feminine gender identity]”

### Human rights violations in public life

Some participants reported being discriminated while being interviewed for visas or having difficulty at immigration counters because the gender status in their citizenship certificates and passports did not match their physical appearance. They reported being cursed by drivers while boarding microbuses in the city. Several participants described difficulty in obtaining rented apartments. *“The owners ask us for higher prices or ask us to vacate the house once they found out our MSM/TG status”.* One of the participants stated,

*“I am dalit* [a group of people of different castes traditionally regarded as untouchables] *and third gender and have experienced double discrimination. I have been called a mad person.”*

According to another participant,

People say that homosexuals are unnatural. They are untrue and copy foreigners.”

Several participants reported that the media portrayed men who have sex with men and third gender as sex workers and prostitutes and did not respect their confidentiality because it published the names of individuals involved in specific incidents.

### Human rights violations in the health care domain

Several participants reported human rights violations in the health care setting. According to one participant,

"*“MSM living with HIV/AIDS experience double discrimination. Health professionals have negative attitudes towards us. The doctors mark their prescription for us as people living with HIV and AIDS* [for those MSM who are infected] *and then tell us that there are no beds for us. We cannot get surgery here. We are sent in circles from one Teku Hospital to Bir Hospital] in the city* [of Kathmandu] *and cannot find anyone to take care of us”*"

A third gender participant stated,

"“*Third genders experience discrimination in the health care setting. Nurses and doctors call us names such as ‘two organs’. When we go to clinics to seek treatment for sexually transmitted diseases we are shown as examples to others that ‘if you engage in unnatural sex acts this will become of you.’ That is why we do not attend the clinics*.”"

Others reported receiving lower quality of care or being denied care as a result of their sexual identity or orientation. One participant reported, *“We are tested in the emergency room when they draw our blood but do not tell us the results of our tests.”*

According to another participant*, “I was inaccurately diagnosed with sexually transmitted diseases when the repeat test showed that I did not have the sexually transmitted disease.”*

Several participants reported: “*being laughed at or not respected … because of our physical appearance”.*

According to a participant,

"*“The health professionals ask ‘How should we treat you since you are not male or female?’ They don’t know us. They don’t want to treat us.”*"

### Human rights violations due to lack of adequate legal protection

Some participants also reported the lack of specific laws related to protecting fair and equitable employment regulations for men who have sex with men, or laws guaranteeing the right to inherit property, and right to an education. Several participants commented on the lack of legal status of marriages for men who have sex with men and third gender and the overall lack of adequate legal protection. According to one participant*,*

"*“We do not have clear laws that will recognize men who have sex with men and third gender as an appropriate category in citizenship certificates.”*"

Another participant stated:

"“*Nepal’s justice is blind. There have been some improvements because metis can now use public restrooms. But they still call me names like randi* [derogatory term for prostitute]”"

### Violence at the hands of authorities

Some participants reported that they had experienced violence at the hands of authorities, while others reported being scolded for carrying condoms in public places. A few participants directly described personal accounts of violence.

According to one participant:

"*A month ago, an officer arrested me and asked ‘Do you have male or female organs.’ He … invaded my privacy by touching my organs to determine the gender. They sometimes touch my breast. This has happened three times.”*"

Another participant described how *“… the authorities brutalized me at the lodge with two* [other] *officers a few months ago at another time.”*

A third participant reported, *“A few months ago I was picked up at night 12 o’clock. They took me away from the city to around 25 kms away. I said I was going to use the bathroom and escaped.”*

A fourth participant recalled,

"*“I was picked up by the authorities at 11 at night near [a neighborhood] about 5 months ago. The van had 4 officers. Two of them were senior officers and the other 2 were subordinates. They asked me for money so that they could fill oil in their van. I did not have the money. They said that they would spare me if I had sex with them. Both officers had oral and anal sex. They used condoms which I provided. Then they dropped me off to my place. I did not file any official complaints.”*"

Some participants recounted incidents that happened several years ago. One participant reported,

"“*I was going with my friend at 10 pm on a winter night around 2 years ago. I remember it as a Wednesday. I was detained. My friend fled away. One of the officers was asleep below. The other officer was drunk. I pleaded for help. He forced to perform oral sex. He did not use condoms. I was disgusted with this.”*"

According to another participant, “*Around 8 years ago the van came, they beat us unconscious and they raped us and left us to die in the fields.”* A similar incident occurring six years earlier was related by another participant.

"*“The authorities imprisoned me and offered to release me only after I had sex with him. Then I was released.“*"

According to another participant, *“I was raped by the authorities 7 years ago when I was 14 years old. There were several officers. I did not report this to anyone.”*

### Mental health consequences

Several participants reported symptoms of anxiety surrounding their daily lives. One participant reported being anxious *“because we are in the wrong body”.* One participant was “*worried about the adverse effects of taking hormones* [to maintain their physical appearance]*, because we are unable to afford to go to Bangkok for sex change operations.”*

According to another participant, “*There is a lot of depression as a lot of people are afraid of being themselves. People don’t know who they are.* “

Several participants described a sense of loneliness and social isolation. According to another participant, *“I had the organs of a boy but the activities of a girl. I was always anxious and liked to live alone … I have contemplated suicide but I didn’t get opportunity to commit suicide. I felt I was a burden on the earth but I don’t have the strength to leave* [this earth].”

### Listing and ranking human rights violations

The list of human rights violation categories generated by each of the daily focus groups is shown in Table 1 and the rankings of the top 3 violations are shown in Table 2. To illustrate our ranking process, on the second day 5 participants ranked police violations as being most important, 2 participants ranked educational violations as being important. Three remaining participants ranked discrimination due to lack of adequate legal protection, violations occurring within the family and health care setting as being most important. Hence this tie for the overall 3rd rank was resolved by examining the second preference of the participants. Three participants ranked discrimination due to lack of adequate legal protection as being 2nd most important (which was ranked # 3 on day 2) as compared to only 2 participants who ranked family violations and none who ranked health in their second preference category. Both the first and last group ranked human rights violations at the household level as being most important. These two groups also ranked the need to address social and economic rights violations and those occurring in the public setting. The second group ranked police torture as being the most important human rights violation that needed to be addressed along with those occurring in educational institutions. All three groups ranked discrimination due to lack of adequate legal protection as being among the top three violations.

**Table 1:** Daily listing of domains of human rights violations among Sexual and Gender Minorities in Nepal

**Day 1**	**Day 2**	**Day 3**
General Discrimination	NA	NA
Education	Educational	Educational
Healthcare	Health	Health
Food, Water & Shelter	NA	NA
Friends	NA	NA
Police	Police	NA
Family	Family	Family
Employment	Employment	Work
Political violence	NA	Police
Media	NA	Media
Lack of adequate legal protection	Lack of adequate legal protection	Lack of adequate legal protection
Housing	NA	NA
Mental problems/Suicide	NA	Anxiety/Sex change
NA	Public	Public
NA	NA	Intimate Partner Violence

NA = Not available

**Table 2:** Daily Ranking of Human Rights Violations among Sexual and Gender Minorities in Nepal

	**Day 1**	**Day 2**	**Day 3**
**1**^**st**^**Priority**	Family and workplace discrimination	Police	Human Rights Violations in Family Life
**2**^**nd**^**Priority**	Lack of adequate legal protection	Educational discrimination	Lack of adequate legal protection
**3**^**rd**^**Priority**	Food water and shelter	Lack of adequate legal protection	Rights violations in Public Places

## Discussion

**Figure 1:**
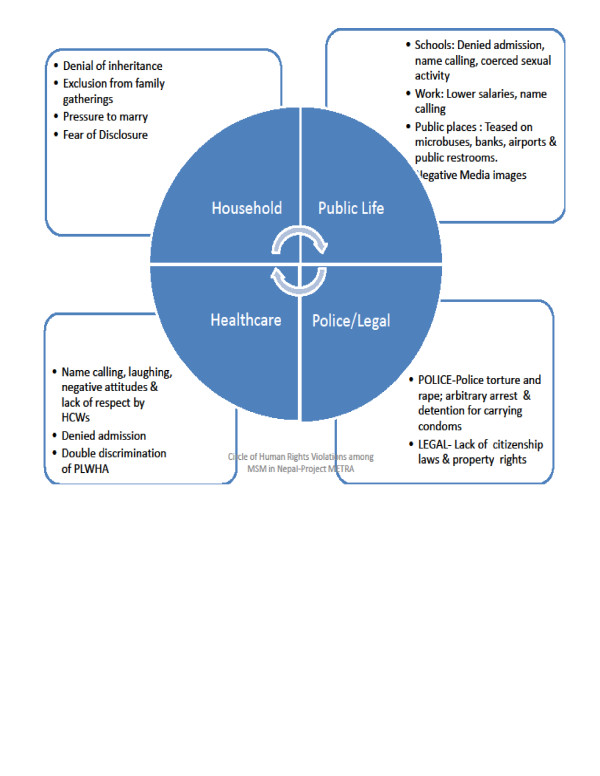
Circle of Human Rights Violations among Sexual and Gender Minorities in Nepal. PLWHA = People Living with HIV AIDS.

The spectrum of participant-reported human rights violations in our study ranged from minor verbal abuse or disrespect such as name calling to egregious violations of human dignity such as rape and coercive sex (Figure 1). Several participants in our study exhibited a remarkable depth of understanding of human rights violations, as illustrated by the use of the term “human rights” to describe their experiences, and the explicit recognition by some participants of the need to specifically address these human rights issues.

The findings should be interpreted in the context of international human rights norms. Nepal has ratified the Convention on Civil and Political Rights, and is also a party to the International Covenant on Economic, Social and Cultural Rights [13,14]. Participant-reported human rights violations such as discrimination against sexual and gender minorities by non-state actors, are not explicitly guaranteed under the traditional concept of international human rights norms for which state actors are held responsible. However, the state has an obligation to protect people from violence, including those committed by non-state actors. These acts of discrimination should also be interpreted in the broader context of *human duties* as reflected in the preamble to the International Covenant on Civil and Political Rights- “*Realizing that the individual, having duties to other individuals and to the community to which he belongs, is under a responsibility to strive for the promotion and observance of the rights recognized in the present Covenant”*[13]*.* Such a broad context of international human rights norms allows one to arrive at a comprehensive understanding of the spectrum of human rights violations experienced by sexual and gender minorities in Kathmandu.

These findings also need to be interpreted in the context of local laws in Nepal. Nepal’s existing civil code of law (Mulki *Ain*) defines rape as “vaginal penetration”, thus providing very little legal recourse for men who have sex with men who experience acts of rape [15]. The legal ambiguity around the prohibition of “unnatural sex” was being used to imprison men who have sex with men in Nepal. In 2007 the Supreme Court of Nepal ended the legal ambiguity by calling sexual and gender minorities’ natural people, therefore ending the potential for the use of the “unnatural sex” clause to be used against sexual and gender minorities [16]. It also issued a decree to issue citizenship certificates with appropriate categorization of gender identity, including a self-identified “third gender.” Although various ministries have expressed support for implementing this third gender category on the national citizenship identification card, the practical realization of these rights has remained difficult due to the lack of implementation and accountability [17]. More recently in 2011 the census in Nepal allowed citizens to identify as male, female, or third gender [17]. The impact of the census was limited, however, because it failed to record third gender citizens along with other meaningful data sets, and there were widespread reports of discrimination during the enumeration. Nonetheless, these changes in the legal environment may have created more awareness which might be partly responsible for the forthright descriptions of personal experiences seen in our study.

These findings should also be interpreted in the context of diverse socio-cultural landscape of Nepal. Some have argued that participant-reported abuse or violence within the family noted in this study fall outside the scope of human rights. Because Nepal is a relatively conservative society, traditional marriage between a man and a woman and subsequent child-raising are still considered essential for maintaining one’s social standing in Nepal, which was until a few years ago an official Hindu state. Any deviation from heterosexuality, explained one of our participants, may be viewed as a Western influence. The exclusion of sexual and gender minorities from cultural events could also reflect an effort on the part of the majority to preserve the traditional “social order” and a form of structural violence. This exclusion appears to hamper the ability of sexual and gender minorities to fully exercise their right to social participation. This social arrangement may ultimately serve to justify or legitimize acts of violence against sexual and gender minorities, and also confer social acceptability on these acts.

Our study also reinforces the synergistic and dual role of social, economic and cultural rights and civil and political rights in the life of sexual and gender minorities in Nepal. The human rights violations identified here encompass various dimensions of social, economic and cultural rights such as the right to food, water and shelter and social inclusion. The participants who identify as third gender reported that the lack of legal recognition on government issued citizenship identification cards has created numerous problems for third gender people. Without proper identification they cannot apply for travel document or open bank accounts [17]. The preferences for legal rights along with changes in their social and cultural life may reflect their desire to ensure that legal rights are an instrument to improve their social, cultural and economic life.

Our results bear similarities and differences with the experiences of sexual and gender minorities in other parts of the world [18]. Difficulty in accessing health care appears to be a common theme for men who have sex with men, around the world [12]. Several participants expressed their reluctance to seek health care because of their experiences at the hands of health professionals, which may further compound their physical and mental health issues. Men who have sex with men in Nepal reported several violations occurring at the household level but, unlike among men who have sex with men in much of sub-Saharan Africa, where men who have sex with men behavior is criminalized, blackmail by families did not appear to be a major issue in our small sample [12]. Sexual and gender minorities in the current study in Nepal cited exclusion from religious ceremonies or cultural functions, paralleling the experience of MSM in Baltimore who report exclusion from church groups. One should be careful in distinguishing the experiences of men who have sex with men who do not identify as third gender with those of people who do identify as third gender, although some studies may have evaluated them together. Some participants in our study, particularly third genders expressed concerns about their physical appearance which made them vulnerable to human rights violations. This is similar to the evidence from other settings such as Brazil that feminine appearing men who have sex with men may be at higher risk of violence due to their distinctive physical difference and expressed gender identity [19].

Our study has limitations. We enrolled a small convenience sample of participants whose responses might reflect the experiences and perceptions of those more closely affiliated with a more outspoken subset rather than more general experience of the broader population. These findings need further verification in a larger population based sample. Self reports may be prone to biases, but we have no reason to suspect the veracity of the accounts. The mutually reinforcing roles of poverty, class, caste, ethnicity cannot be easily disentangled from that of sexual orientation and/or gender identity, as reported by one of our *dalit* [a group of people of different castes traditionally regarded as untouchables] participants who described the experience of *“double discrimination”* [term used by a participant]. Although human rights are interdependent and indivisible, we purposefully asked participants to rank various human rights violations to understand the relative importance of these violations on their daily lives. Some of these categorizations may not be mutually exclusive, and the effect of one human rights violation on the realization (or lack thereof) of other human rights could not be disentangled. The concurrent description of the exposure to human rights violations and the mental health issues of anxiety, depression, loneliness and previous suicidal thoughts suggest a potential association that requires systematic research to further define and quantify.

## Conclusions

Despite these limitations, our findings have potential policy and research implications. Our research indicates that sexual and gender minorities in Nepal experienced a wide range of human rights violations. Future efforts should enroll a larger and more systematic sample of participants to determine frequency, timing, and/or intensity of exposure to rights violations, and estimate the population-based impact of these rights violations on specific health outcomes. Such efforts will inform interventions to reduce exposure to violations or the risk of subsequent adverse physical and mental health outcomes.

### Description of terminology

The description of terminology for sexual and gender minorities in Nepal is as below

Metis, singarus or kothis are effeminate homosexual men.

Dohoris are gay or bisexual men who do not necessarily present as feminine

Tas are the sexual partners of metis and dohoris who see themselves as masculine and mostly act like heterosexual males.

Hijras (sometimes called eunuchs) are those who are born biologically male and wish to be female, and some undergo castration and join the hijra community, a traditional religious sect.

Third gender- is a term used in Nepal to describe: people who are biological males who have "feminine" gender identity/expression and/or people who are biological females who have "masculine" gender identity/expression.

## Abbreviations

(MSM), Men who have sex with men; (MSM/TG), Men who have sex with men and third genders; (PLWHA), People living with HIV/AIDS.

## Competing interests

The authors declare that they have no competing interests.

## Authors’ contributions

SS designed and implemented the study, synthesized the analysis plan, analyzed the data and led the writing of the article. SB Pant assisted with the conduct of the study and the writing of the article. SD assisted with the conduct of the study and the writing of the article. SB Pokhrel assisted with administrative support, data analysis and writing of the article. LCM design and implemented the study, helped synthesize the analysis plan and assisted with the writing of the article. All authors helped to conceptualize the study, interpret findings and review drafts of the article. All authors read and approved the final manuscript.

## Pre-publication history

The pre-publication history for this paper can be accessed here:

http://www.biomedcentral.com/1472-698X/12/7/prepub
